# Effect of DPSS Nd: YAG (532 nm) and diode (980 nm) laser treatment on fluoride uptake in dentin following silver diamine fluoride application: An in vitro study

**DOI:** 10.1007/s10103-026-04855-y

**Published:** 2026-03-27

**Authors:** Howaida Elsegaey, Nagy Abdulsamee, Ahmed Abbas Zaky, Omnia Hamdy

**Affiliations:** 1 Cairo University, Giza, Egypt; 2 Misr University for Science and Technology, Cairo, Egypt

**Keywords:** Fluoride therapy, Silver diamine fluoride, Nd:YAG, Diode Laser irradiation

## Abstract

Fluoride treatment has long been recognized as an important method for treating dental decay. Silver Diamine Fluoride (SDF) treatment has emerged as a low-cost and simple approach, combining silver’s antimicrobial properties with fluoride’s caries-inhibiting activities. The present study compares the effects of two lasers, diode-pumped solid-state frequency-doubled Nd: YAG (DPSS, 532 nm) and diode (980 nm), on fluoride absorption in dentin when used with SDF. Following treatment with 38% SDF, the prepared dentin specimens were exposed to radiation for 3, 5, and 10 s using either a frequency-doubled Nd: YAG laser (532 nm, 1 W, free-space beam, 3 mm diameter) or a diode laser (980 nm, continuous wave) at 1 W and 3 W power with a 300 μm optical fiber scanned tangentially across the surface at a speed of 1 mm/s. The equivalent energy densities for 1 W and 3 W diode irradiation were approximately 1,414.7 J/cm² and 4,244.1 J/cm², while for Nd: YAG exposure, it ranged from 42.4 to 141.4 J/cm². Evaluation methods included scanning electron microscopy (SEM) to assess dentinal tubule openness and energy-dispersive X-ray (EDX) analysis to quantify fluoride uptake. Statistical analyses included the Kruskal-Wallis test and Mann-Whitney comparisons, with Spearman’s correlation coefficient used to examine relationships between image analysis and EDX results. Results indicated that the combination of SDF treatment with laser irradiation significantly increased fluoride uptake and decreased open dentinal tubules compared to SDF treatment alone (overall P < 0.001). The SDF + diode 3 W group exhibited the highest mean fluoride wt% and the lowest mean% open tubules (significant compared to SDF alone, P < 0.05). The fd-Nd: YAG 10 s group exhibited fluoride levels comparable to diode 3 W. There was a significant negative correlation (ρ = -0.78, P < 0.001) between fluoride wt% and percentage of open tubules. Laser irradiation with Diode at 3 W or fd-Nd: YAG Laser-assisted SDF treatment enhances tubule blockage and boosts surface fluoride retention in a laboratory context. These findings support the use of lasers to optimize the effectiveness of SDF in caries prevention and dentinal tubule sealing. However, more in-vivo and thermal safety research is required before making a clinical recommendation.

## Introduction

Dental caries is a highly prevalent non-communicable disease that affects people of all ages, from early childhood to elderly age. Despite advances, this disease remains prevalent in socioeconomically poor regions, although its occurrence has decreased in developed countries during the last decade [1, 2]. Dental caries is the chemical breakdown of tooth hard tissues caused by acid generated by the bacterial decomposition of sugar. This acid decreases the pH around the tooth, causing mineral loss, enamel and dentin demineralization, and deterioration of dentin’s type I collagen [3, 4]. Silver diamine fluoride (SDF) has been considered a powerful anti-caries agent for many years [5]. This novel approach shows promise in stopping cavitated dentine carious lesions and preventing new caries from developing [6]. SDF therapy emerges as a simple, painless, non-invasive, and cost-effective intervention, necessitating minimal equipment and support [7].

SDF’s versatile applications extend beyond early childhood caries to include root caries management, pit and fissure caries prevention, secondary caries inhibition, remineralization of hypomineralized teeth, infected root canal treatment, and desensitization of hypersensitive teeth [8]. SDF is typically available as a 38% solution containing 255,000 ppm silver and 44,800 ppm fluoride ions [9]. It uses silver’s antibacterial abilities to battle cariogenic biofilms, while fluoride aids in remineralization and protects teeth from demineralization. Furthermore, SDF deactivates proteolytic peptidases and slows dentine collagen breakdown [10]. It efficiently prevents caries without compromising dental pulp integrity or causing dental fluorosis. While indirect pulp capping with SDF often produces little or no inflammatory pulpal response, direct administration to the tooth pulp may result in pulp necrosis [11].

Researchers investigated the combination of laser irradiation with SDF therapy to prevent tooth fractures during endodontic treatment and prevent the development of root caries by increasing fluoride uptake in dentine [12]. Several studies investigated the influence of the laser-fluoridation approach combined with laser application on fluoride release and caries prevention [13]. The observations indicate that the approach improves fluoride adherence to enamel and integration to hydroxyapatite, especially when combined with topical fluoride treatments. The combination method increases the production of fluor-hydroxyapatite and calcium fluoride (CaF₂) deposits on the enamel surface, resulting in a persistent fluoride reservoir [14]. While laser treatment alone improves enamel’s acid resistance, the combination of laser and fluoride is the most effective technique for caries prevention [15].

Studies have highlighted that lasers can enhance fluoride uptake by enamel, dentin, and in the management of root caries. This increased fluoride absorption, facilitated by laser technology, underscores the evolving role of lasers in augmenting traditional preventive and therapeutic approaches in dental practice [16]. Zhao et al. [17] reported that using a 9.3 μm carbon dioxide laser and SDF improved enamel demineralization and acid resistance. The researchers attributed the increased resistance to inherent changes in the enamel structure. They also noticed less bacterial adherence to the enamel after laser treatment. In SDF-treated dentin, Mei et al. [18] concluded that the diode laser (810 nm) group had higher fluoride uptake than the Nd: YAG laser group. But compared to the diode and Nd: YAG laser groups, the CO2 and Er: YAG laser groups revealed much greater fluoride uptake levels. Moreover, some researchers have investigated the use of low-power lasers to control and prevent demineralization processes in dental structures, with positive results for increases in enamel microhardness [19, 20].

Although previous research has examined the potential benefits of combining light-based therapies with SDF application, a recent systematic review by [21] found no clinical studies that demonstrated a significant additional benefit of laser irradiation when combined with SDF for caries prevention. Earlier investigations demonstrated that using light-curing devices, such as blue LEDs at 430–490 nm and irradiances of 800-1,200 mW/cm², to activate SDF and enhance its photochemical interaction with dental tissues did not significantly improve caries arrest in primary teeth [22]. These results highlight the need for additional research to comprehend the potential benefits and underlying processes of combining laser irradiation with traditional caries-prevention treatment methods.

The efficacy of different lasers in facilitating fluoride uptake is mainly related to the tissues they target [23]. Diode and Nd: YAG lasers minimize water absorption while specifically targeting pigmented tissues such as haemoglobin and melanin. Dentin can be subtly stained with silver diamine fluoride (SDF), which may eventually improve the absorption of Nd: YAG laser energy. The complicated dynamics of dental treatments are highlighted by the interaction of tissue properties, laser wavelengths, and SDF staining [24]. The current study aims to examine and compare the effects of two laser types (980-nm diode laser and 532-nm diode-pumped solid-state frequency-doubled (DPSS Nd: YAG) on fluoride uptake after SDF application. The objective is to investigate how laser irradiation can improve fluoride absorption in dentin, potentially increasing the efficacy of SDF treatment.

## Methods

In the present study, Fluoride uptake and distribution within dentin samples were evaluated using scanning electron microscopy (SEM) and energy dispersive X-ray (EDX) analysis. Statistical analysis was carried out using Kruskal-Wallis testing, followed by Mann-Whitney comparisons between the different experimental groups. Additionally, Spearman’s correlation coefficient was employed to explore the relationship between the SEM images and EDX results, providing valuable insights into fluoride absorption patterns in dentine following the specified treatments.

### Samples collection and preparation

**Fig. 1:**
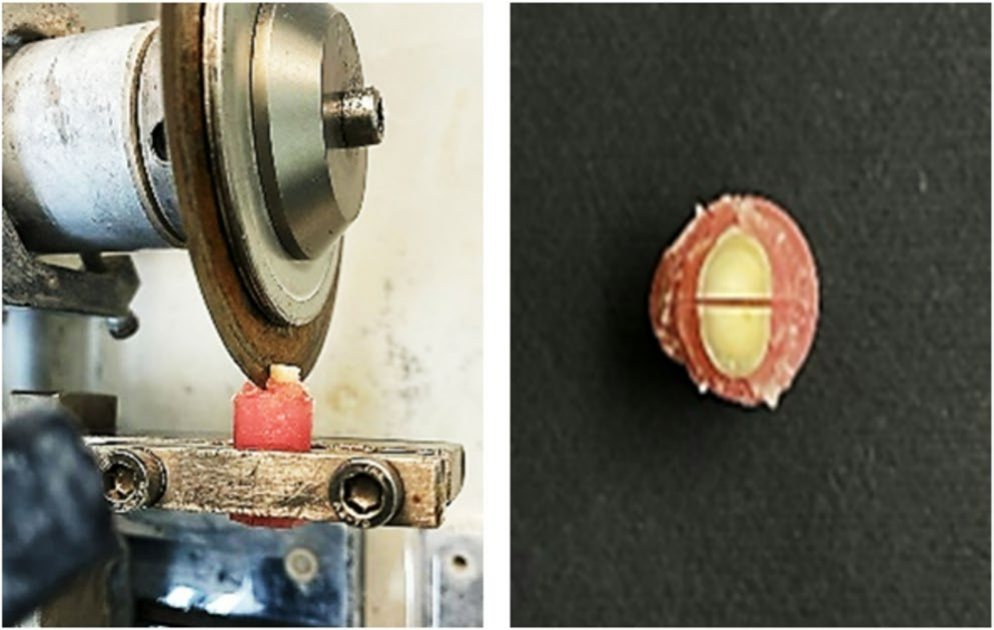
Sample preparation steps

Seventy recently extracted premolar teeth, originally intended for orthodontic purposes, were carefully selected for this study. The inclusion criteria stipulated that the teeth must exhibit no defects, microcracks, erosion, caries, or restorations. These teeth were subsequently stored in distilled water to maintain their integrity until processing. Each tooth was sectioned at cemento-enamel junction using the Isomet 4000 Buehler Linear Precision cutting system.

(Buehler, Germany) to obtain standardized dentine slices with a thickness of 3 mm. The slices were further bisected using diamond blade under water to ensure precision and consistency in preparation as illustrated in Fig. 1.

Prior to laser treatment, the smear layer on all dentin slices was removed using 18% ethylenediaminetetraacetic acid (EDTA) for 1 min, then rinsed with distilled water and gently air dried. 38%-SFD (e-SDF, Kids-e- dental LLP, Mumbai, India) was applied with a microbrush and gently rubbed over the dentin surface for 2 min per the manufacturer’s guidelines. Any excess solution was removed by gentle air drying with a 3-in-1 syringe.

#### Experimental Groups

The prepared dentine slices were divided into seven experimental groups (*n* = 10 each).

1. Group 1 (Control): no treatment.
2. Group 2 (SDF only): treated with SDF solution.
3. Group 3 (SDF + Diode 980 nm, 1 W): treated with SDF solution followed by diode laser irradiation at 1 W (Group 3) or 3 W (Group 4) for 3 s, fiber core 300 μm; scanned at ≈ 1.0 mm/s; exposure time = 3 s (total scan duration for the specimen). (Irradiance ≈ 1,414.7 W/cm²; estimated radiant exposure per point ≈ 424.4 J/cm².)
4. Group 4 (SDF + Diode 980 nm, 3 W): treated with SDF solution followed by diode laser irradiation at 1 W (Group 3) or 3 W (Group 4) for 3 s, fiber core 300 μm; scanned at ≈ 1.0 mm/s; exposure time = 3 s (total scan duration for the specimen). (Irradiance ≈ 4,244.1 W/cm²; estimated radiant exposure per point ≈ 1,273.2 J/cm².)
5. Group 5 (SDF + DPSS Fd-Nd: YAG 532 nm, 1 W, 3 s): SDF followed by Nd: YAG (532 nm) irradiation in contact mode, power = 1 W; spot diameter = 3 mm; exposure time = 3 s (irradiance ≈ 14.14 W/cm²; radiant exposure ≈ 42.42 J/cm²).
6. Group 6 (SDF + DPSS fd-Nd: YAG 532 nm, 1 W, 5 s): same as Group 5 with exposure time = 5 s (radiant exposure ≈ 70.7 J/cm²).
7. Group 7 (SDF + DPSS fd-Nd: YAG 532 nm, 1 W, 10 s): same as Group 5 with exposure time = 10 s (radiant exposure ≈ 141.4 J/cm²).

After the respective treatments, the dentine slices were carefully examined for fluoride and silver content analysis.

### Laser irradiation protocols

**Fig. 2:**
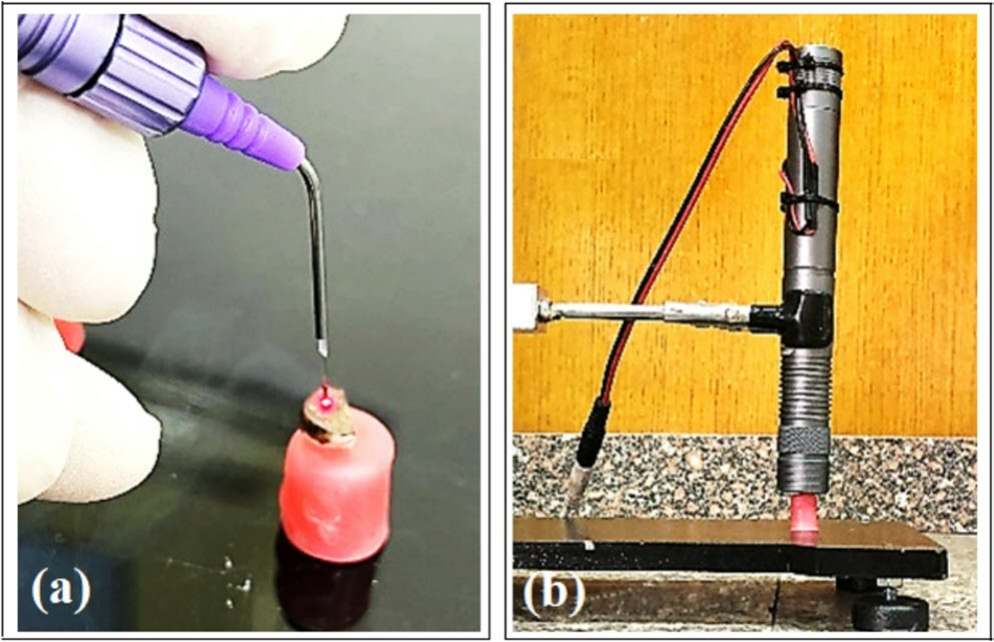
Representative images of the laser delivery systems utilized in this study: (**a**) 980 nm diode with 300-µm fiber tip adjacent to dentin surface (the image shows fiber tip and sample holder); (**b**) 532 nm fd-Nd: YAG free-space contact irradiation with 3 mm spot diameter. (Only the two panels shown were used to provide method illustrations.)

Laser irradiation was applied to the surface of specimens in groups 3 and 4. A continuous wave (CW) diode laser device (Zolar Technology & Mfg Co. Inc., Canada) emitting light at a wavelength of 980 nm was utilized. A fiber optic probe of 300 μm, scanned manually over the dentin surface at a controlled speed of approximately 1 mm/s. The diode laser (980 nm) was applied in contact mode with a non-initiated fiber tip standing directly on the dentin surface throughout irradiation. Contact mode provided a stable beam profile and constant energy delivery. The operator maintained consistent tangency and movement across the samples, though minor human variation is acknowledged (see Fig. 2). The fiber optic probe was manually held perpendicular to the surface and moved longitudinally in a uniform scanning motion across the entire exposed surface. The energy densities were calculated based on the power settings and beam characteristics, resulting in approximately 1414.7 J/cm² for the 1 W diode laser and 4244.1 J/cm² for the 3 W setting, both over a 3-second exposure duration.

Groups 5, 6, and 7 were subjected to irradiation with a DPSS fd-Nd: YAG laser (Laserlands, China) emitting light at 532 nm and a power of 1 W (as specified by the manufacturer). The laser was operated in contact mode with varying exposure times of 3 s, 5 s, and 10 s for groups 5, 6, and 7, respectively. The laser beam was directed perpendicularly onto the dentin surface with an estimated spot diameter of 3 mm (area ≈ 0.0707 cm²). This resulted in an irradiance of approximately 14.14 W/cm². The radiant exposures delivered were 42.42 J/cm², 70.7 J/cm², and 141.4 J/cm² for the 3-, 5-, and 10-second exposure durations, respectively).

### Fluoride uptake and morphological assessment

For the analysis of the dentine surface morphological structure and elemental composition, the specimens were meticulously characterized utilizing a Scanning Electron Microscopy (SEM) equipped with Energy Dispersive X-ray (EDX) analysis (FEI Quanta 3D 200i). This equipment operates under conditions of an accelerating voltage ranging between 20 and 30 kV with a resolution for Gun at 1 nm. For SEM analysis, the samples were mounted on aluminum studs using carbon adhesive tape and coated with gold to enhance conductivity. Furthermore, the fluoride and silver content within the dentine samples was quantitatively measured for each specimen using the EDX analysis. For each specimen, the SEM operator obtained an overview at low magnification before using a systematic grid technique to choose three typical areas inside the observed area (center and two randomly chosen peripheral spots within the yellow ROI) for EDX. During acquisition, the technique is automated and has no regard for group allocation in order to reduce selection bias.

The SEM image analysis was carried out using “ImageJ” software (version 1.53a, NIH, USA). Each micrograph’s entire image area (in µm²) was automatically calculated. Open dentinal tubules were detected using thresholding and particle analysis methods, and their cumulative area was calculated. As demonstrated in Eq. (1), the percentage of open dentinal tubules was computed by dividing the total open tubule area by the total image area and multiplying by 100 [25]. All measurements were automated using ImageJ’s image processing stages, with no manual counting involved.

1 $$\mathrm{Open}\;\mathrm{dentinal}\;\mathrm{tubules}\;\%\;=\frac{\mathrm{Total}\;\mathrm{area}\;\mathrm{of}\;\mathrm{opened}\;\mathrm{dentinal}\;\mathrm{tubules}\;(\mathrm{\mu{m}})}{\mathrm{Total}\;\mathrm{image}\;\mathrm{area}\;(\mathrm{\mu{m}})}\times100$$

To determine if surface fluoride content is associated with tubule occlusion, we compared the specimen-level mean (%) from EDX to the specimen-level mean percentage of open tubules from ImageJ. EDX measures elemental fluoride at the sampling sites, whereas image analysis measures tubule openness as an area fraction; a negative correlation would indicate that greater fluoride deposition is related with increased tubule blocking. This method enabled consistent and automated measurement, minimizing the potential of observer bias and allowing for precise assessment of treatment effects on dentinal permeability.

### Statistical analysis

Data were analyzed using the Shapiro-Wilk test for normality. Non-parametric tests were applied: the Kruskal-Wallis test for group comparisons and the Mann-Whitney U test for pairwise analysis. Spearman’s correlation coefficient was used to assess relationships between image analysis and EDX results. Statistical significance was set at *P* < 0.05. Statistical evaluation was performed using the SPSS statistical package (version 23 (2015), IBM Co. USA).

## Results

### Fluoride uptake analysis (EDX results)

**Fig. 3:**
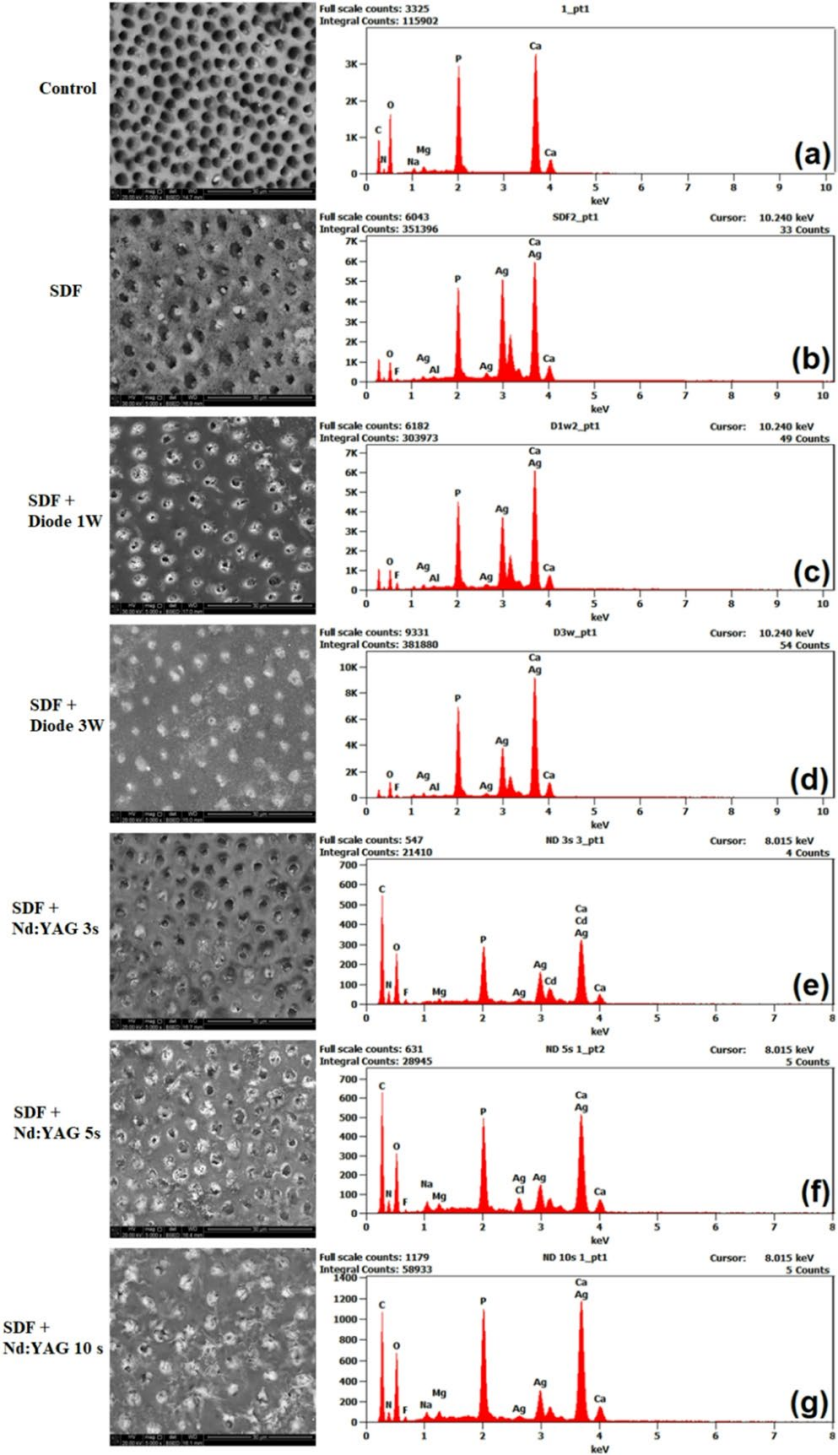
Representative SEM micrographs (×5,000; scale bar = 30 μm) and corresponding EDX elemental maps of dentin surfaces after different treatments. (**a**) control sample, (**b**) SDF sample, (**c**) 1-W diode laser irradiated sample, (**d**) 3-W diode laser irradiated, (**e**) 3-sec Nd: YAG irradiated sample, (**f**) 5-sec Nd: YAG irradiated sample, (**g**) 10-sec Nd: YAG irradiated sample

Examples of the obtained SEM images and EDX spectra for each sample group are presented in Fig. 3. The obtained SEM images at a magnification of x5000 revealed distinct characteristics of dentinal tubule surfaces across different treatment groups. Each specimen was first examined at low magnification to observe the predominant surface morphology. Following this initial survey, five image fields were captured at the study’s standardized magnification (×5000) from five distinct and evenly distributed locations across the surface. This systematic approach ensured that the selected fields were representative of the entire specimen and were not chosen selectively based on surface appearance. All imaging was performed by an operator who was blinded to the group allocation, thereby eliminating any potential bias during field acquisition.

As shown in Fig. 3, the untreated control group had a typical dentin surface that included multiple open dentinal tubules of uniform circular appearance and well-defined peritubular margins. SDF treatment alone resulted in partial precipitation of surface deposits; however, several tubules remained visible, indicating incomplete occlusion. In contrast, laser-assisted SDF treatments resulted in significant surface changes. The number and diameter of open tubules in the SDF + diode 1 W group decreased significantly, with scattered granular deposits covering the surface. Increasing the diode laser power to 3 W provided a denser, more continuous layer of fused and recrystallized material, nearly fully sealing the tubule openings. The degree of tubule closure in the fd-Nd: YAG (532 nm) groups was proportional to the exposure time. After 3 s of irradiation, only minor reduction of tubule orifices was detected, whereas 5 s of exposure resulted in more visible bonding of peritubular areas. The 10 s group had a compact surface layer with few apparent tubule openings, similar to the morphology exhibited in the diode 3 W group.

**Fig. 4:**
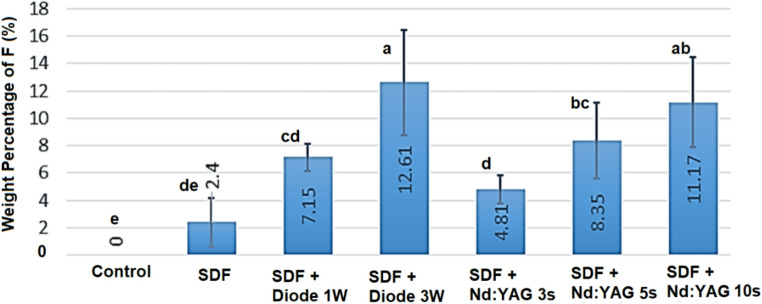
The mean ± SD of fluoride weight% (F wt%) assessed by EDX for each group (*n* = 10). Different letters above bars indicate statistically significant differences between groups (Mann-Whitney U tests with Bonferroni correction), while groups sharing the same letter are not substantially different (*P* ≥ 0.05)

**Fig. 5:**
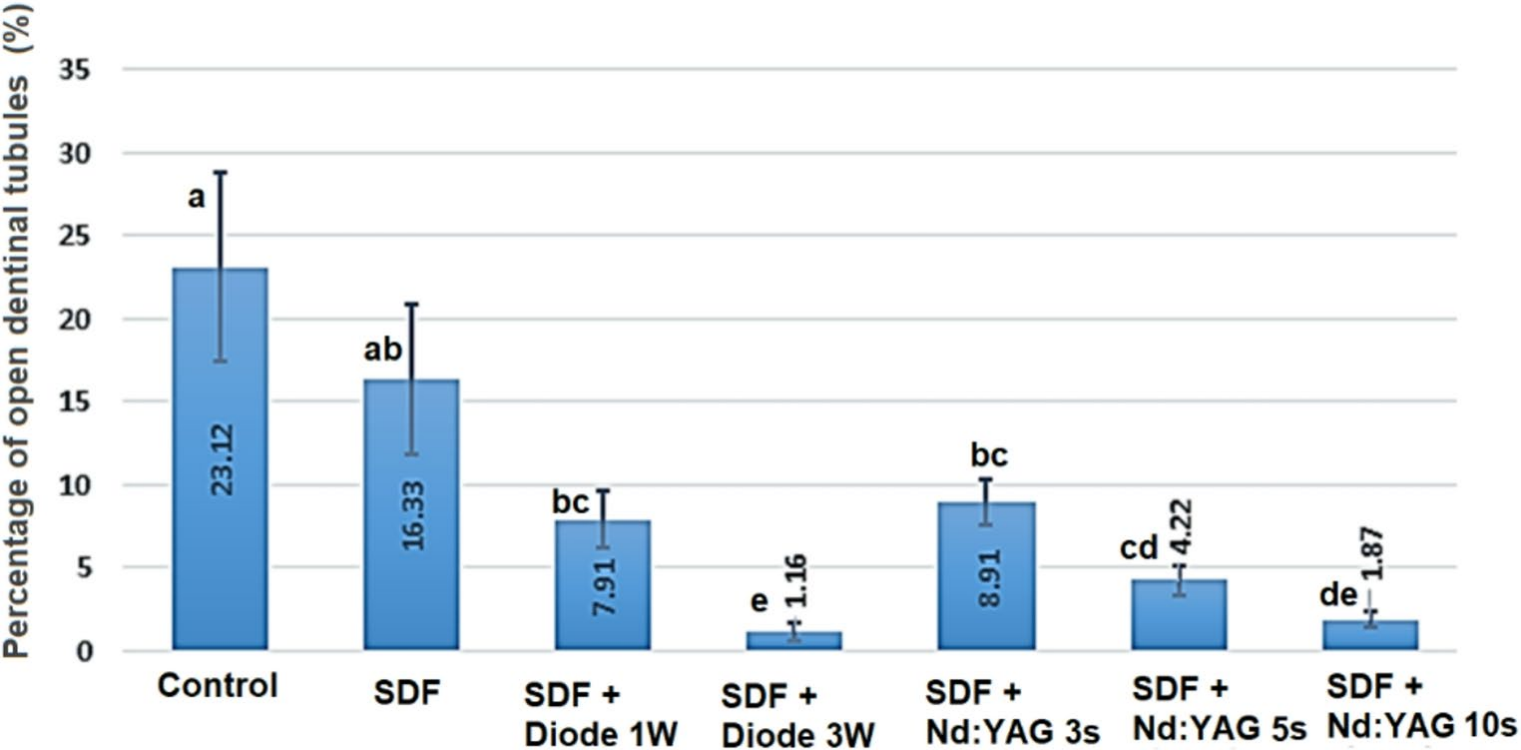
Percentage of open dentinal tubules by group (mean ± SD; *n* = 10). Different letters above bars indicate statistically significant differences between groups (Mann-Whitney U tests with Bonferroni correction), while groups sharing the same letter are not substantially different (*P* ≥ 0.05)

The quantitative EDX analysis are illustrated in Figs. 4 and 5. Figure 4 shows the mean fluoride content (% wt F) of dentin surfaces after treatment. The control group had no fluoride signal (0.00 ± 0.00%), while the SDF-only group showed a slight increase (2.40 ± 1.30%), indicating SDF’s fundamental absorption capacity. When SDF was used in conjunction with laser irradiation, fluoride uptake increased significantly. The SDF + diode 3 W group had the highest fluoride deposition (12.61 ± 3.82%), significantly higher than the SDF + diode 1 W group (7.15 ± 2.94%) and the SDF-only group (*p* < 0.05). Fluoride uptake in the Nd: YAG laser groups gradually increased with exposure duration, from 4.81 ± 1.72% (3 s) to 8.35 ± 2.96% (5 s) and 11.17 ± 3.31% (10 s). The 10 s Nd: YAG group achieved fluoride levels comparable to the diode 3 W group, demonstrating the efficacy of sustained exposure in enhancing ion penetration. All laser-assisted SDF treatments significantly increased fluoride incorporation compared to SDF alone (*p* < 0.05). The results indicate that both laser type and irradiation parameters—power and exposure duration—are crucial in optimizing fluoride uptake in dentin.

Quantitative image analysis revealed that the proportion of open tubule area decreased significantly (*p* < 0.05) in all laser-treated groups compared to the SDF-only and control groups (see Fig. 5; Table 1). The diode 3 W and Nd: YAG 10 s groups had the lowest open-tubule percentages, which matched their maximum fluoride uptake levels determined by EDX. This morphological evidence backs up the synergistic effect of laser irradiation and SDF therapy in increasing dentinal tubule sealing and reducing permeability.

**Table 1:** Mean ± SD values of fluoride uptake (%) and percentage of open dentinal tubules (%) for all treatment groups. Different letters indicate statistically significant differences between groups (*p* < 0.05; Kruskal–Wallis and Mann–Whitney tests)

Group	Fluoride Uptake (%) ± SD	Open Dentinal Tubules (%) ± SD
Control	0.00 ± 0.00 (e)	23.12 ± 5.00 (a)
SDF	2.40 ± 2.40 (de)	16.33 ± 4.50 (ab)
SDF + Diode 1 W	7.15 ± 1.20 (cd)	7.91 ± 1.00 (bc)
SDF + Diode 3 W	12.61 ± 3.80 (a)	1.16 ± 0.60 (e)
SDF + Nd: YAG 3s	4.81 ± 1.50 (d)	8.91 ± 1.20 (bc)
SDF + Nd: YAG 5s	8.35 ± 2.30 (bc)	4.22 ± 0.80 (cd)
SDF + Nd: YAG 10s	11.17 ± 3.30 (ab)	1.87 ± 0.50 (de)

### Spearman’s correlation between image analysis results and EDX results

**Fig. 6:**
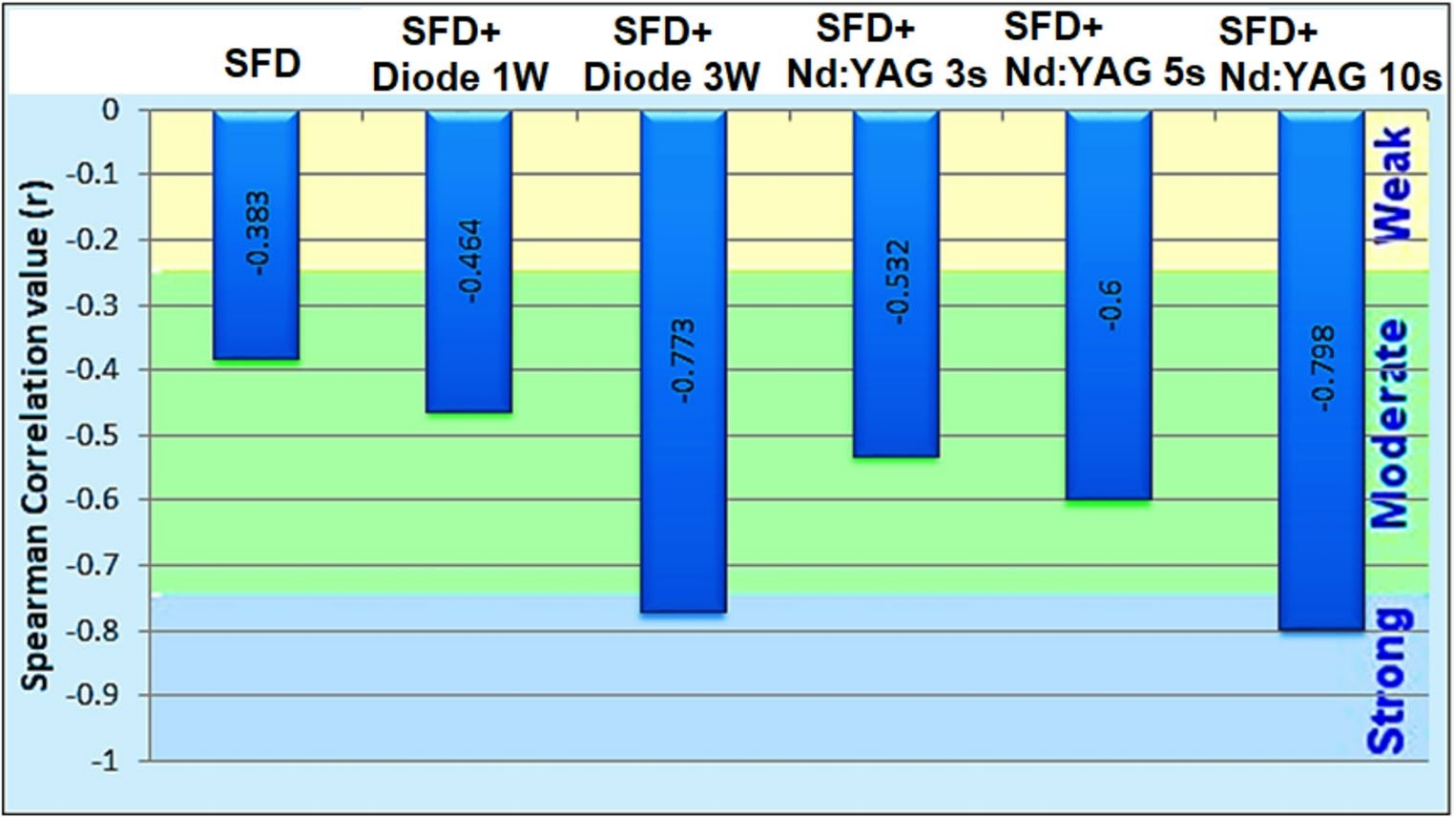
A scatter plot demonstrating the association between fluoride weight% (EDX) and the percentage of open dentinal tubules (ImageJ). The figure shows the Spearman correlation coefficient (ρ) and the p-value

Figure 6 illustrates the results of the Spearman’s correlation test between the open dentinal tubule percentage calculated using ImageJ and the fluoride weight% (wt%) acquired from EDX measurements.

From the results presented in Fig. 6, we can conclude the following:

- There was a moderate negative correlation between the percentage of opened dentinal tubules and the weight% of F Wt % in groups SDF, SDF diode 1 W, SDF Nd 3 s, and SDF Nd 5 s, and these correlations were statistically significant (P-value < 0.05) in SDF and SDF diode 1 W and highly significant in SDF Nd 3 s and SDF Nd 5 s (P-value < 0.001).
- There was a strong negative correlation between the percentage of opened dentinal tubules and the weight% of F Wt % in groups SDF diode 3 W and SDF Nd 10 s and these correlations were statistically highly significant (P-value < 0.001).

## Discussion

This study provides valuable insights into the effectiveness of laser-assisted fluoride therapy in enhancing fluoride uptake in dentin treated with SDF via examining different laser irradiation protocols following the application of SDF. Group 3 received diode laser irradiation at 1 W for 3 s after SDF treatment, while Group 4 underwent diode laser irradiation at 3 watts for 3 s post-SDF application. Groups 5, 6, and 7 were treated with Nd: YAG laser irradiation at 1 W for 3, 5, and 10 s. These distinct durations of laser exposure were carefully chosen to investigate the effects of different power, irradiance and radiant exposure on the fluoride uptake in dentine following SDF treatment, contributing valuable insights to the study’s evaluation of the efficacy of laser-assisted fluoride therapy. All laser treatment were performed by the same operator to minimize variability. The diode and Nd: YAG lasers were utilized in our laboratory setting under multiple variable settings. Consequently, the direct comparisons between modalities are exploratory since each modality’s parameters were altered independently. Stronger conclusions on the impact of the variable parameter on fluoride uptake and tubule blocking can be derived from in-modality comparisons (e.g., diode 1 W vs. 3 W; Nd: YAG 3 s vs. 10 s).

The results clearly demonstrate that combining SDF with laser irradiation significantly improves fluoride penetration and reduces the percentage of open dentinal tubules compared to SDF alone. These effects were more pronounced when higher laser power (diode laser) or longer exposure times (Nd: YAG laser) were employed. A laser-only group without SDF application was excluded from this investigation because the primary goal was to evaluate the possible effect of laser irradiation on the performance of SDF in boosting fluoride uptake and dentinal tubule occlusion. As a result, the study concentrated on optimizing laser parameters to improve the efficacy of SDF therapy. While the isolated thermal or structural effects of laser exposure on dentin may provide useful mechanistic insights, such investigations are beyond the scope of this work and could be addressed in future studies aimed specifically at characterizing laser-tissue interactions independent of fluoride application.

It should be mentioned that the comparisons in this study included changing the exposure time in the Nd: YAG laser group and the power in the diode laser group. The operational constraints and modes of the various laser systems in our lab provided the basis for this strategy. Despite being distinct factors, power and time were both altered within their respective laser groups to investigate their effects on dentinal tubule sealing and fluoride uptake. Consequently, direct comparisons between the diode and Nd: YAG groups should be regarded as exploratory, and results within each laser type should be interpreted independently. Because of intrinsic losses related to fiber coupling and beam divergence, it is necessary to keep in mind that the actual power reaching the dentin surface may be less than the nominal output. To provide more meaningful inter-modal comparisons, future research should employ standardized energy density (J/cm²) values across laser types rather than only matching nominal power or exposure time, as energy dose is the parameter most directly associated with tissue response. The irradiance and radiant exposure parameters presented in the Methods were computed based on measured power and beam/fiber geometry. These estimated quantities are estimates of the nominal energy delivered to the specimen surface under the specified conditions. In practice, beam divergence, coupling efficiency and slight differences in tip-to-surface geometry can all have an impact on the amount of energy that reaches tissue. Because these variables can alter thermal and photochemical effects, they should be considered when evaluating inter-study variations or translating these parameters into clinical practice.

The enhanced fluoride uptake observed in this study is likely attributed to the structural modifications induced by laser irradiation. Laser energy is known to alter the crystalline structure of dentin and reduce the resistance of the smear layer, allowing for deeper penetration of fluoride ions [26]. The 3-W diode laser group exhibited the highest fluoride uptake and the most effective tubule sealing, followed closely by the Nd: YAG laser group with 10 s of exposure. This suggests that optimizing laser parameters, such as power settings and exposure duration, is critical for achieving the best outcomes. These findings align with previous studies that have reported the benefits of laser-assisted fluoride treatments. For instance, Mei et al. [18] demonstrated that CO₂ and Er: YAG lasers significantly enhanced fluoride uptake in SDF-treated dentin compared to Nd: YAG and diode lasers. Similarly, Bahrololoomi et al. [27] found that CO₂ lasers were more effective in promoting fluoride uptake in enamel than diode lasers. Such results support the growing body of evidence that laser technologies can improve fluoride retention in dental tissues, contributing to remineralization and caries prevention. Table 2 lists variations in laser types, wavelengths, exposure settings of different laser systems that have been used to improve fluoride uptake in dental hard tissues compared to our utilized parameters.

**Table 2:** Comparison of previous studies evaluating laser-assisted fluoride uptake and dentin modification with the present study

Ref	Laser Type	Wavelength (nm)	Mode (CW/Pulsed)	Power (W)	Spot / Fiber Diameter (mm)	Exposure Time (s)	Estimated Irradiance (W/cm²)	Substrate & Fluoride
[13]	Nd: YAG	1,064	Pulsed	1.5	--	--	--	Dentin + AgF
[18]	CO₂ / Er: YAG / Nd: YAG / Diode	10,600 / 2,940 / 1,064 / 810	Pulsed / CW	1–3	0.8–1.0	1–10	700–3,800	Dentin + SDF
[27]	Diode	810	CW	2	0.6	30	707	Enamel + Topical fluoride
[28]	Er, Cr: YSGG	2,780	Pulsed	0.25–0.5	0.8	10	~ 250–500	Enamel + APF
Present study	Diode	980	CW	1 / 3	0.3	3 (scanned)	1,414.7 / 4,244.1	Dentin + SDF
	fd-Nd: YAG	532 nm	CW	1	3.0	3 / 5 / 10	42.4 / 70.7 / 141.4	Dentin + SDF

A substantial negative correlation between fluoride uptake and the percentage of open dentinal tubules was observed in this study, underscoring the dual benefits of laser irradiation. By facilitating fluoride penetration and reducing tubule permeability, laser-assisted treatments not only enhance fluoride retention but also reduce dentinal hypersensitivity. This dual effect makes laser-assisted SDF therapy a promising option for managing high-risk patients, including those with caries susceptibility or dentinal hypersensitivity. These findings support earlier research that shown how laser irradiation might alter the morphology of the dentinal surface, facilitating deeper fluoride ion penetration and fixing [28]. Because fluoride reinforces the tooth structure by generating fluorapatite, which is more acid-resistant than hydroxyapatite, increasing the tooth’s resistance to demineralization and possible caries development, increased fluoride concentration in dentin is of therapeutic importance [29].

In terms of controlling dentinal hypersensitivity, our results show that laser-assisted SDF treatment has a superior tubule sealing impact than SDF alone. The observed reduction in open dentinal tubules shows that laser energy improves the occlusion process, most likely by thermally induced dentin surface modifications such as protein denaturation and structural reconfiguration of the smear layer. These modifications may limit fluid flow within the tubules, which is thought to be the major cause of sensitivity. This is consistent with prior research showing that laser irradiation can increase dentin resistance to external stimuli by narrowing or sealing tubule orifices. Beyond enhanced fluoride uptake, the use of diode and Nd: YAG lasers may provide additional therapeutic value in the form of decreased dentinal permeability, making this combined treatment appealing for individuals with hypersensitivity [30].

Low-level laser energy interacts with dentin’s natural components (chromophores and hydroxyapatite), producing photothermal and photobiomodulatory effects. These effects gently warm the surface and reorganize the mineral lattice while avoiding damaging ablation. This regulated reorganization increases the mobility and binding of fluoride ions inside the dentin matrix. Furthermore, laser light may stimulate local non-thermal photobiological processes that promote mineral nucleation and ionic exchange. The final impact is the creation of stable, acid-resistant deposits such as fluorapatite and calcium fluoride-like compounds, which minimize dentin permeability. Siddig et al. [31] demonstrated that using a particular diode laser and sodium fluoride gel treatment efficiently sealed dentinal tubules with homogenous, fluoride-rich crystals while preserving tissue structural integrity.

Collagen is gently thermally softened and contracted by the 980 nm diode laser, which is primarily absorbed by water and organic materials. This process facilitates surface fluoride deposition and surface compression. On the other hand, silver pigments, like those in SDF, are the main target of the 532-nm Nd: YAG laser. Deeper fluoride penetration results from this reaction, which starts a photoactivated subsurface ionic exchange. Fluoride incorporation can be safely increased without causing surface damage by extending the Nd: YAG exposure time to 10 s. In comparison to other systems like Er: YAG, both diode and Nd: YAG systems have been demonstrated to be successful when employed at low fluence with fluoride, resulting in denser mineral deposits and more complete dentinal tubule occlusion [32]. The comparison of laser-based and light-based activation emphasizes the importance of energy density. Crystal et al. [33] reported that LED curing light enhanced SDF penetration marginally but did not result in the same structural or compositional alteration as laser activation, indicating that sufficient photon energy is required to induce ionic diffusion and mineral reorganization. The sub-ablative diode and Nd: YAG lasers used in the current work deliver this energy safely, resulting in measurable fluoride boosts while preserving tissue viability [33].

While SDF is already recognized as a cost-effective treatment for caries, combining it with laser irradiation offers additional advantages by enhancing its efficacy. Moreover, the minimally invasive nature of this approach makes it an attractive option for broader clinical applications. However, this study has some limitations. The in vitro design, while controlled, may not fully simulate clinical conditions, where factors such as saliva, biofilm, and patient variability could influence treatment outcomes. Additionally, the long-term effects of laser-enhanced fluoride therapy on dentin integrity and the durability of fluoride retention need further investigation.

Our results showed that when diode laser irradiation and SDF were applied together, fluoride uptake increased and dentinal tubule apertures decreased. It is crucial to remember that the mechanism behind these observations has not yet been completely clarified, even if this points to a possible improvement at the microstructural level. According to earlier theoretical assessments [34], laser irradiation may cause surface alterations that are both chemical and physical and make it easier to incorporate fluoride. It is uncertain to ascertain if the effects that have been observed are additive or synergistic without assessing the effects of laser irradiation alone (without SDF). Therefore, more research separating the effects of each treatment component is required.

Despite our in-vitro Nd: YAG regimen (especially 10 s) significantly improved tubule closure and fluoride uptake, prolonged contact irradiation at these radiant exposures could cause elevated temperatures in dentin and pulpal tissues in vivo. Previous studies indicate that increases in pulpal temperature above ~ 5–7 °C may risk pulpal injury [35], so physicians should assess in-vitro efficacy results alongside thermal safety data before clinical translation. Future research should incorporate thermocouple or thermography readings during irradiation, as well as an investigation into pulpal temperature rise on extracted teeth with simulated pulpal circulation, in order to define clinically safe exposure limits. Pulsed regimes, reduced duty cycles, or intermittent irradiation may produce equivalent surface effects at a lower thermal load and should be investigated. Moreover, the reported results’ medical significance still needs more research through carefully planned clinical studies. It is important to note that better caries resistance or arrest in vivo are not necessarily implied by higher fluoride uptake in vitro. Clear proof of better clinical results should be used to promote the inclusion of laser technology in caries treatment protocols, particularly in communities with limited access to care. To date, no clinical studies have definitively established a significant increase in benefit from laser irradiation when combined with SDF treatment. Consequently, future research should focus on validating the proposed findings through clinical trials to better understand the long-term benefits and safety of laser-assisted fluoride therapy.

Given that SDF is mostly utilized in groups with limited access to care, such as children and the elderly, it is imperative to assess the practical implications of including laser irradiation into standard SDF treatment protocols. In these situations, a demonstrated and clinically significant improvement in results must be used to support any new procedure that extends the course of therapy and increases costs. Although our in-vitro data demonstrate that combined diode laser and SDF treatment increases fluoride uptake and decreases dentinal tubule openings, the findings’ therapeutic significance has not yet been established. Laser irradiation should be used cautiously until better therapeutic benefits are proven through extensive trials, especially in areas with little infrastructure and resources. Exploring its efficacy in diverse patient populations and under varying oral conditions will provide a more comprehensive evaluation of its clinical utility.

## Conclusions

This in-vitro study demonstrated that combining SDF application with diode (980 nm) and frequency-doubled Nd: YAG (532 nm) laser irradiation significantly increased fluoride uptake and reduced dentinal tubule openness compared to SDF alone. Both laser modalities increased fluoride incorporation by controlling photothermal alteration of the dentin surface, resulting in tubular closing with little visible surface damage. The 3 W diode and 10-second Nd: YAG irradiation parameters had the strongest influence, demonstrating that laser power and exposure duration are both important for optimizing treatment outcomes. These results indicate that laser activation can improve the physicochemical interaction between fluoride ions and dentin, resulting in increased mineral stabilization and surface protection. While the results suggest that laser-assisted SDF therapy has promising potential for increasing dentinal resistance and decreasing permeability, further studies on thermal safety, durability under pH cycling and mechanical wear, and clinical performance for caries prevention and dentin hypersensitivity reduction is required before translation to clinical practice. Moreover, the conclusions are confined to in-vitro conditions, therefore, additional in situ and clinical studies are needed to evaluate the long-term durability, biocompatibility, and safety of this method, as well as to develop standardized irradiation parameters for practical dental application.

## Data Availability

The datasets used and/or analyzed during the current study are available from the corresponding author on reasonable request.
